# Evolutionary game theory and simulations based on doctor and patient medical malpractice under government regulation

**DOI:** 10.1038/s41598-023-44915-9

**Published:** 2023-10-25

**Authors:** Lin Song, Zhenlei Yu, Juntao Fang, Qiang He

**Affiliations:** 1 Tianjin University of Traditional Chinese Medicine, Tianjin, 301617 China; 2 Information Ministry of Library, Qilu University of Technology, Jinan, 250100 China

**Keywords:** Public health, Quality of life

## Abstract

Physicians-patients are the two crucial participants in medical malpractice. The government, as the central authority responsible for addressing medical malpractices, plays a pivotal role in this matter. Guided by governmental agencies, its regulations, administrative orders, and policies serve as the primary governance mechanisms to address medical malpractice, providing an effective means to balance the doctor-patient relationship and foster social harmony and stable development. A doctor-patient evolutionary game model developed based on the principles of information asymmetry and finite rationality. The study explores the strategic decision-making process of these two players within the context of medical malpractice. Through the manipulation of various parameters, the model's evolutionary equilibrium strategy is demonstrated using Vensim PLE Version 6.4 simulation. The findings reveal that government regulation, patient cognition, and the benefits associated with standardized medical practices are the pivotal factors influencing the doctor-patient evolutionary game system under government regulation. It is possible to mitigate medical malpractice through adjusting relative weights of differing strategic options, adding penalties for unlawful conduct, and normalizing malpractice charges on the basis of physicians' income from standardized practice. To effectively address medical malpractice, proposed measures include adjusting the regulatory framework, reasonably determining the strength of regulations regarding medical practitioners' illegal practices and patient medical malpractice behavior, diversifying regulatory approaches, establishing comprehensive physician–patient management systems for information to resolve medical malpractices.

## Introduction

As China's medical and healthcare system reform deepens, the issues related to healthcare services are becoming increasingly prominent, severely impacting the mutual trust, respect, and understanding between doctors and patients, and have garnered significant attention from society as a whole.

This study utilized the Web of Science core collection database to search for relevant studies published from October 10, 2012, to October 10, 2022, with the keywords "Medical Negligence" OR "Medical Malpractice." The search identified 1956 studies. Due to the multifaceted nature of medical malpractice, this study places particular emphasis on researching medical malpractice in the context of legal proceedings, claims, adverse events, and misconduct. To ensure the accuracy and objectivity of the analysis, unrelated literature such as editorials, proceedings papers, and letters were excluded. Only research and review articles were included. Ultimately, 1235 studies were included in the analysis.

**Figure 1:**
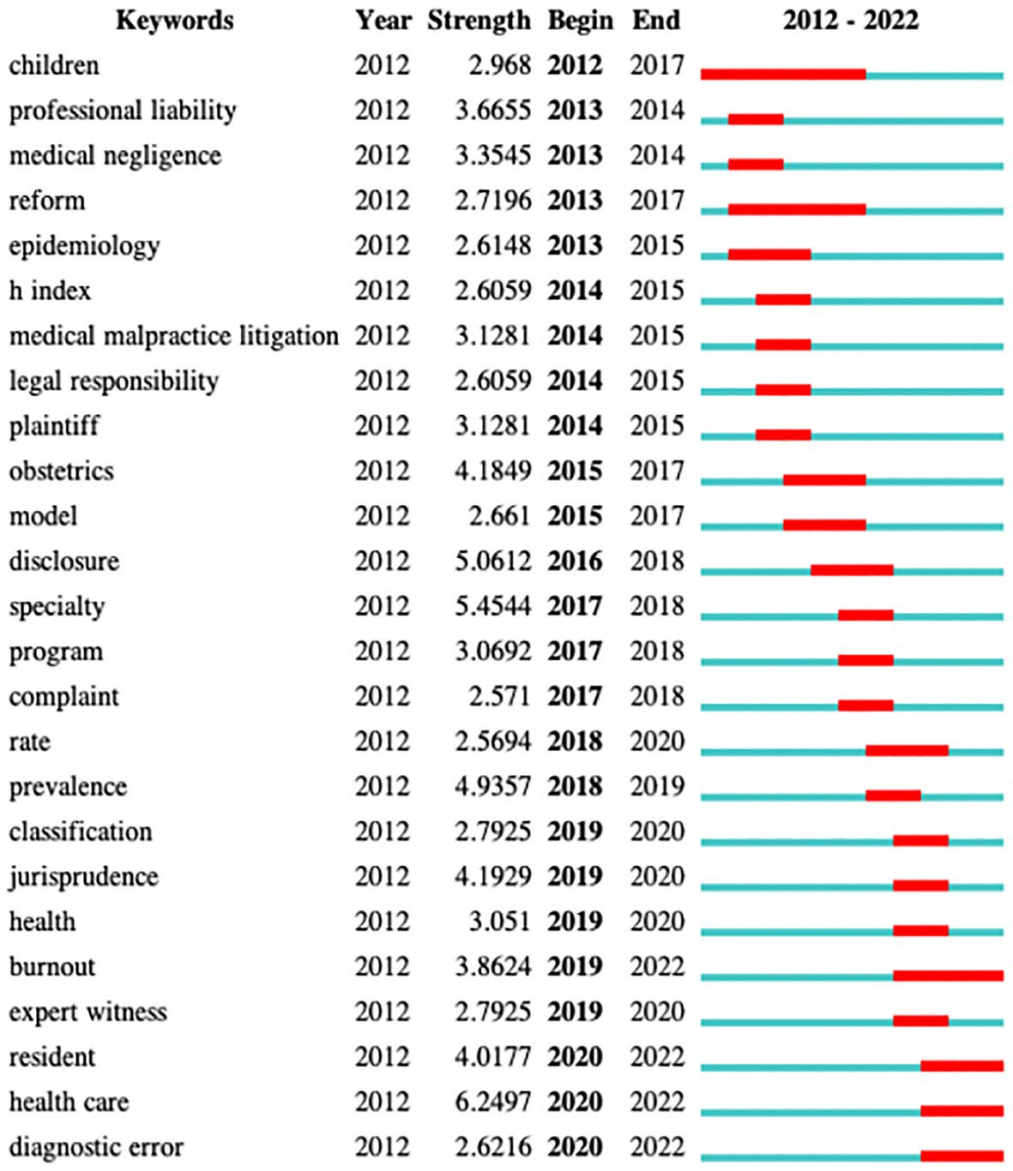
The prominent medical malpractice research keywords identified by the word map generated from 2012 to 2022 research articles.

**Figure 2:**
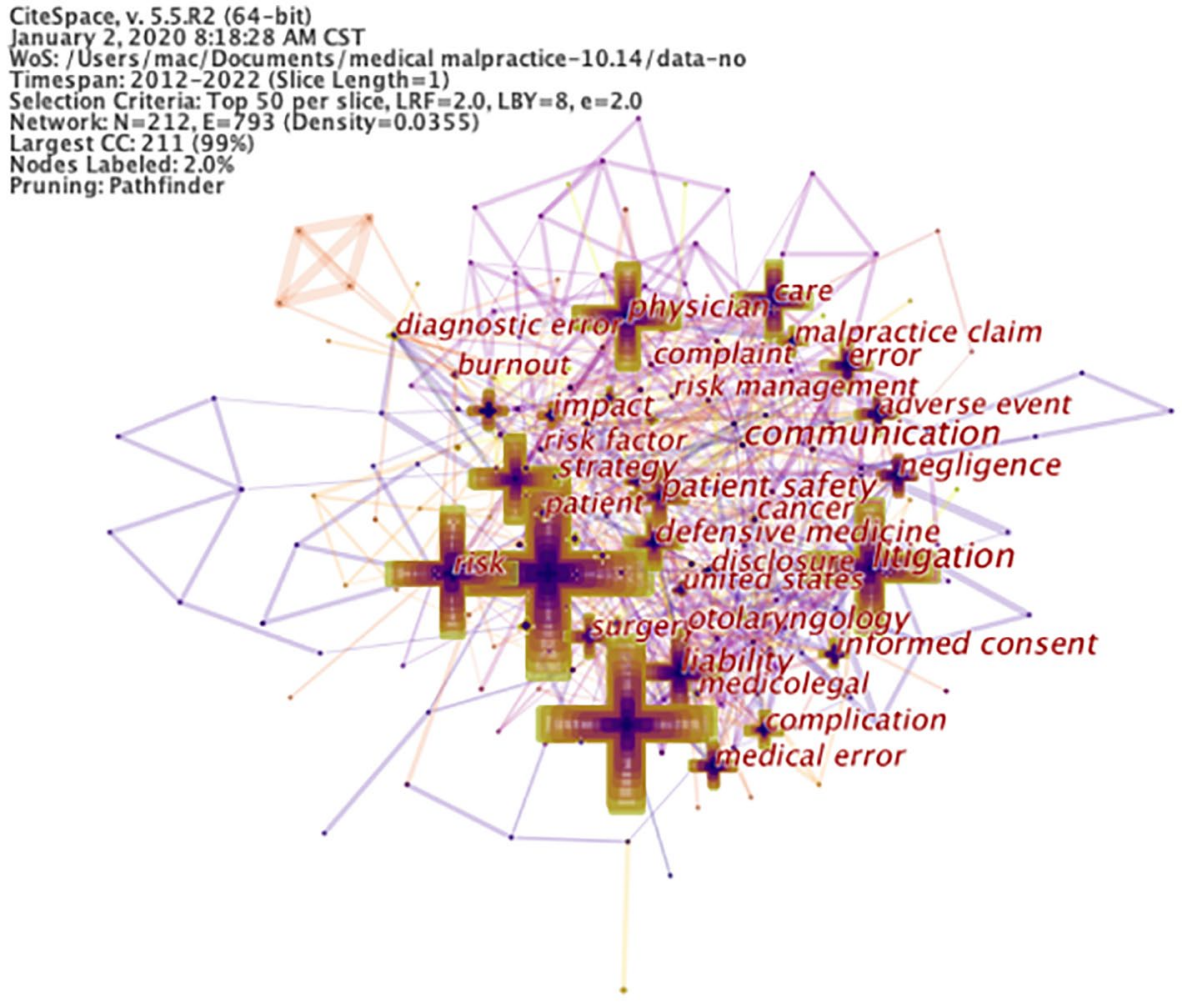
Keyword map of medical malpractice from 2012 to 2022.

Recently, doctor-patient disputes have become increasingly frequent in China. Through the co-occurrence of keywords in the research literature on medical malpractice (as depicted in Fig. 1) and the statistical analysis using Citespace V5.5 R2 of the related data, it can be seen that from 2012 to 2022, there were 31 keywords with a frequency higher than 30^3^. Among these, the keywords "physician", "patient safety", and "medical error" occurred up to 89 times. The year of occurrence was earlier, and the centrality was high (Fig. 2), reflecting that "physician", "patient safety", and "medical error" were the primary focus of medical malpractice research during the period^1,2^. Moreover, keywords such as "risk", "claim", "litigation", "cost", and "mortality" reflected that the research content of medical malpractice mainly revolved around compensations and the improvement of relevant laws^3^. For example, the keywords "satisfaction", "risk management", "association", "attitude", and "complaint" reflected that medical malpractice was primarily patient-centered. As such, patient satisfaction and attitude, as well as effective communication, are crucial for resolving medical malpractice. These factors were also associated with a strong management foundation^4,5^. The keywords such as "system", "model", "information", "time", and "network" reflected that medical malpractice research has begun to expand into the areas of modeling and information systems^6,7^.

Upon reviewing the available research results, it is evident that there are few studies on medical malpractice under government regulation both domestically and abroad. The concept of government-regulated medical malpractice has not been consistently defined. Few studies consider the impact of government regulation on the evolution of medical malpractice, nor do they consider the impact of different combinations of strategies employed by medical parties, such as standardized or illegal practices, cooperation or conflict strategies, on patient strategies. Furthermore, there is a lack of a clear definition of disputes between doctors and patients under government regulation.

Therefore, the author posits that disputes between doctors and patients under government regulation refer to the regulation of prohibitions and restrictions on specific behaviors of both doctors and patients for the purpose of safeguarding patient health, hygiene, and safety. Administrative means are employed to address the complexity of medical services and the ambiguity of causality, as well as the externalities and information asymmetry that exist during disputes between physicians and patients about the adverse consequences and causes of diagnosis and treatment^8–11^. At the same time, the game model plays an important role as one of the essential analytical tools in the review of medical malpractice research methods. Pan Hongwei and Yu Hui constructed a model of the doctor-patient interest game, arguing that the "failure of Nash equilibrium" is the crux of the doctor-patient game conflict^12^. Zeng et al. theorized the doctor-patient relationship based on game theory and concluded that the occurrence of doctor-patient disputes is mainly due to medical information asymmetry and communication barriers between doctors and patients^13,14^. In recent years, the evolutionary game strategy of doctor-patient disputes has often been used to study the evolutionary development process of two or three parties from the perspectives of standardized practice, due diligence, cooperation strategy, doctor-patient disputes or non-doctor-patient disputes. This approach has also been explored more in the research objects^15–19^.

Moreover, previous studies have primarily focused on the analysis of doctor-patient game theory, which can contribute to the reduction of medical malpractice. Nevertheless, there is little scholarly discussion of how physicians' choices between two strategies impact the medical behavior of their patients. For example, the evolutionary games by Antoci et al. on doctors' defensive medical choices and on doctors' choices of insuring against malpractice liability have not been extensively studied^20–22^. There is also limited research that has used quantitative approaches to investigate the selection of two diverging strategies for doctors, the strategies of healthcare behaviors of patients, and how to manage the evolutionary game of controlling factors that impact medical malpractice in the direction of a stated goal. For instance, the evolutionary games by Antoci et al. on patients’ defensive medical choices^23,24^ offer valuable insights into this area^25^.

The primary characteristics of this study are as follows:

1. Grounded in the context of bounded rationality, this study conceptualizes medical malpractice as a dynamic game characterized by ongoing choices and variations. Evolutionary game theory is employed to examine the phenomenon of medical malpractice within the framework of government oversight. Consequently, we formulate an evolutionary game model that delineates the interactions between physicians and patients under government regulation. The findings of this study shed light on the equilibrium behaviors adopted by healthcare practitioners and patients in the context of government regulation.
2. This study delves into the selection of strategies aimed at stabilizing the dynamics between physicians and patients during instances of medical malpractice under government oversight. Additionally, it assesses the equilibrium and stability of the interactions between physicians and patients in such scenarios. To further enhance our understanding, we employed Vensim PLE version 6.4 to simulate the model, varying parameters like physician revenue and the intensity of government regulation. This simulation provides a more vivid representation of the evolutionary trajectory of the interactions between physicians and patients in the context of medical malpractice under government oversight. Consequently, it underscores the importance of implementing measures to mitigate the complexities inherent in doctor-patient relationships, thereby ensuring effective governance.

## Results

To validate the earlier conclusions, this study examines the stability strategies employed by physicians and patients during physician–patient disputes by conducting simulations using Vensim evolution. When assigning parameters, akin to numerous prior studies, this research adopts direct assignment methods based on previous research and real-world considerations. It is essential to emphasize that the primary focus of this study is not precise parameter assignment but rather the comprehension of the trend changes in the probability of strategy selection by medical practitioners and patients during doctor-patient disputes. Consequently, we acknowledge a certain margin of acceptable error in parameter assignment.

**Figure 3:**
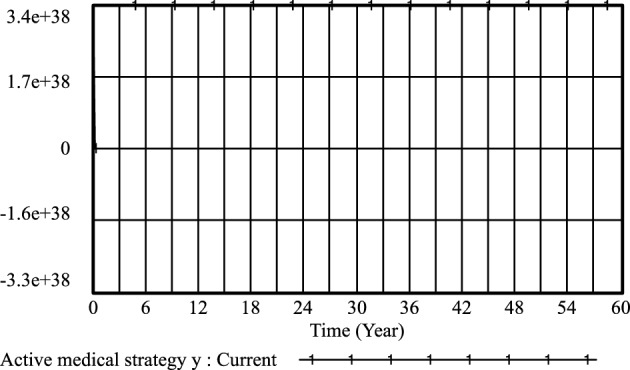
Active medical strategy y evolutionary diagram (Li Chaoran as an example).

**Figure 4:**
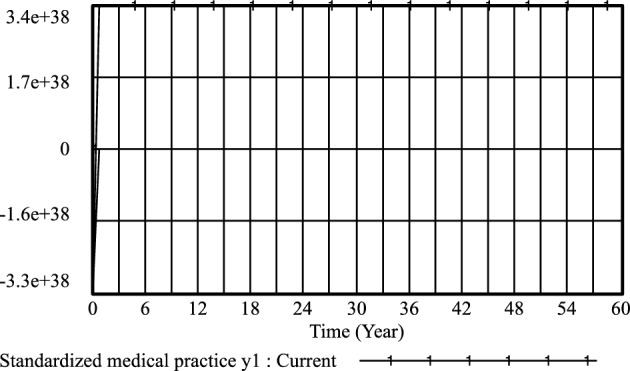
Standardized medical practice *y*_*1*_ evolutionary diagram (Li Chaoran as an example).

**Figure 5:**
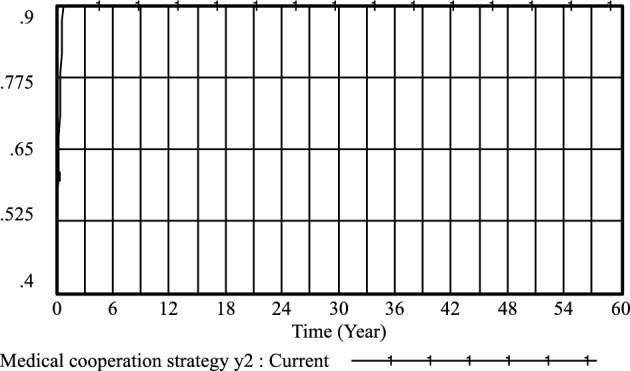
Medical cooperation strategy *y*_*2*_ evolutionary diagram (Li Chaoran as an example).

**Figure 6:**
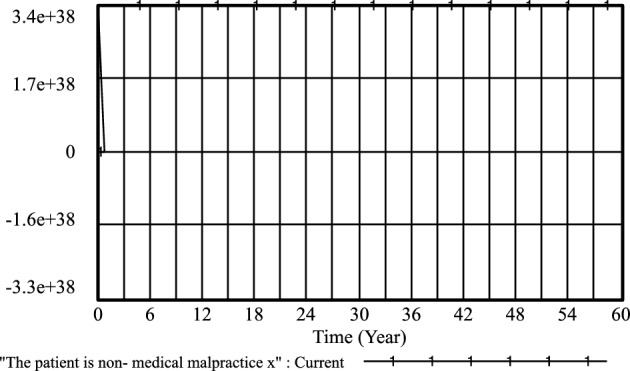
The patient is non-medical malpractice *x* evolutionary diagram (Li Chaoran as an example).

**Figure 7:**
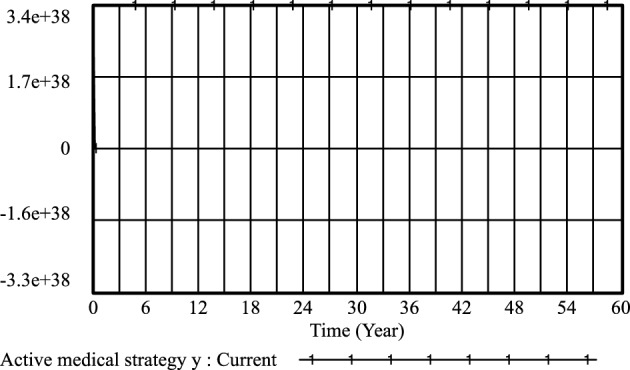
Active medical strategy *y* evolutionary diagram (Liu Jusheng as an example).

**Figure 8:**
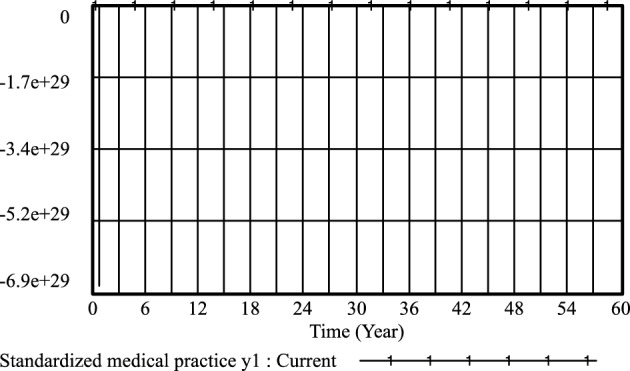
Standardized medical practice *y*_*1*_ evolutionary diagram (Liu Jusheng as an example).

**Figure 9:**
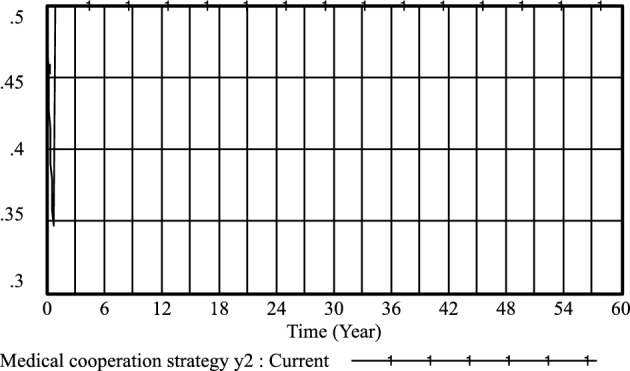
Medical cooperation strategy *y*_*2*_ evolutionary diagram (Liu Jusheng as an example).

**Figure 10:**
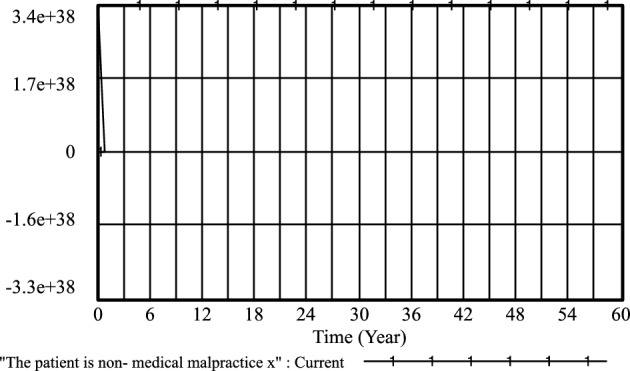
The patient is non-medical malpractice *x* evolutionary diagram (Liu Jusheng as an example).

**Figure 11:**
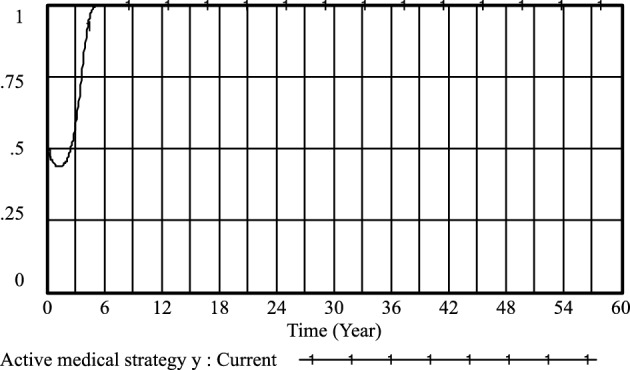
Active medical strategy* y* evolutionary diagram (Liu J as an example).

**Figure 12:**
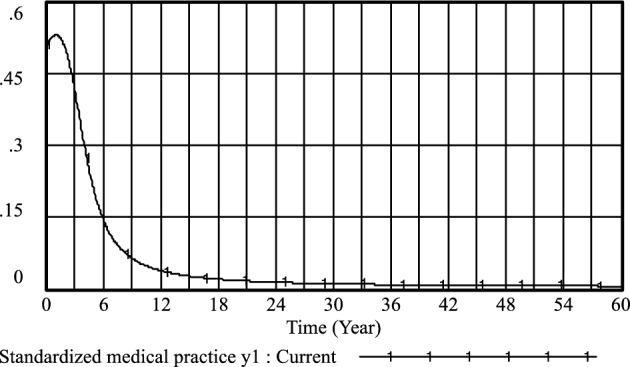
Standardized medical practice* y*_*1*_ evolutionary diagram (Liu J as an example).

**Figure 13:**
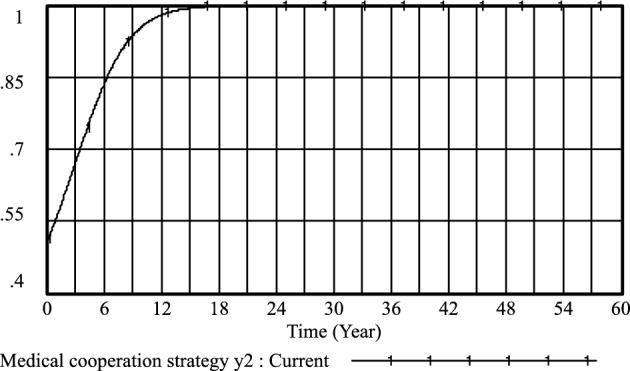
Medical cooperation strategy *y*_*2*_ evolutionary diagram (Liu J as an example).

**Figure 14:**
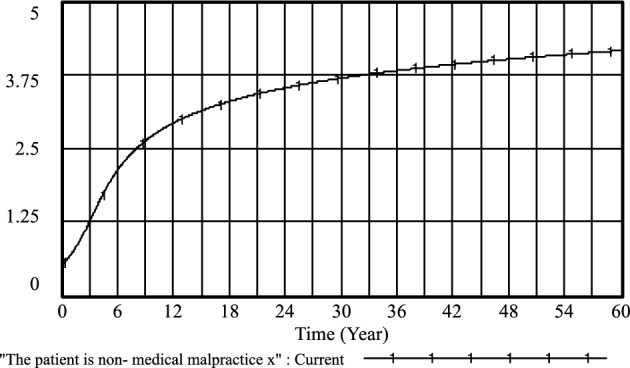
The patient is non-medical malpractice *x* evolutionary diagram (Liu J as an example).

Furthermore, the cooperation and conflict costs associated with medical prescriptions have been thoroughly investigated by previous researchers, including Li^26^, Liu^27^, and Liu^28^. Their findings, as depicted in Figs. 3, 4, 5, 6, 7, 8, 9, 10, 11, 12, 13, and 14, have been subjected to comprehensive analysis. It has been ascertained that Liu's parameters, setting the cooperation cost at 0.2 and the conflict cost at 0.4, represent the most suitable values. Similarly, the benefits and costs associated with patient cognition have been examined by researchers such as Guo^29^, Wu^30^, and Shu^31^. This collective analysis has led to the determination of the optimal values for patient cognition. It is worth noting that the specific steps for establishing the benefits and conflict costs of cooperation strategies align consistently with these findings and need not be reiterated.

On the basis of Liu J’ study results, this research sets the cooperation cost to 0.2 and the conflict cost to 0.4. Combining this with the actual situation, we iteratively determine the specific parameters as follows: *k* = 0.4*S*_*1*_ = 0.5, *S*_*2*_ = 0.5, *O*_*1*_ = 0.2, *O*_*2*_ = 0.4, *O*_*3*_ = 0.3, *O*_*4*_ = 0.2, *PE*_*1*_ = 0.5, *PE*_*2*_ = 0.8*L*_*1*_ = 0.1, *L*_*2*_ = 0.3, *H* = 0.6, *D* = 0.6, *M* = 0.9, *E* = 0.3, *R*_*1*_ = 0.5, *R*_*2*_ = 0.3, *U*_*1*_ = 0.5, *U*_*2*_ = 0.7. By adopting this approach, we aim to establish a robust model that aligns with both previous research and real-world scenarios.

### Evolutionary simulation research

By adjusting the income of doctors when they regulate practice under government regulations, the following results are obtained: current: *PE*_*1*_ = 0.4; current 1; *PE*_*1*_ = 0.6 and current 2:*PE*_*1*_ = 0.8.

**Figure 15:**
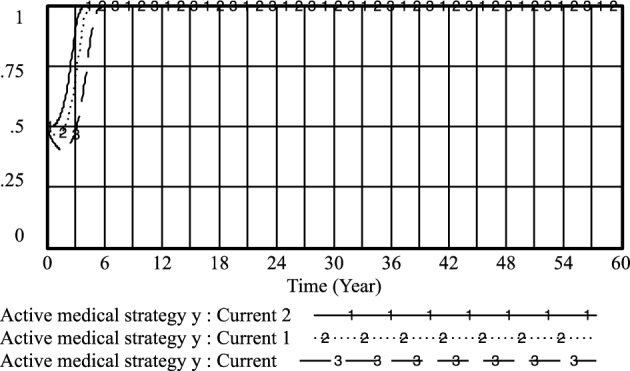
Effect of *PE*_*1*_ on physician positive strategy selection.

**Figure 16:**
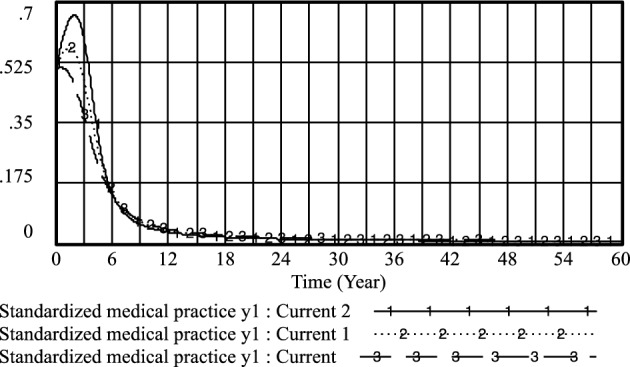
Effect of *PE*_*1*_ on physician standardized medical practice strategy selection.

**Figure 17:**
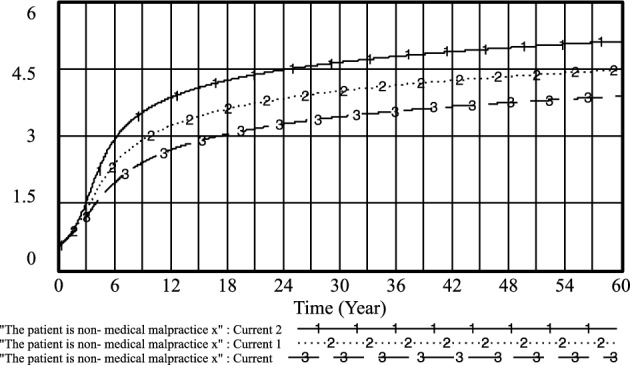
Effect of *PE*_*1*_ on patients' selection of non-medical strategy.

As illustrated in Figs. 15, 16, and 17, it becomes evident that as doctors derive greater benefits from adhering to standardized practices, their likelihood of opting for proactive strategies increases, as does the speed with which they make this choice. Conversely, the probability of patients refusing treatment from such doctors also rises with the associated increase in benefits for doctors.

However, it's noteworthy that the probability of standardized practice peaks in the short term as *PE*_*1*_ increases, but then experiences a rapid decline. This indicates that when doctors' returns from choosing standardized practice surpass or equal the income from illegal practice, the likelihood of doctors selecting standardized practice initially rises but subsequently diminishes significantly. This suggests that effective monitoring and regulation of doctors' income related to standardized practice can encourage medical practitioners to adopt standardized practice strategies more promptly. However, simply increasing the income associated with standardized medical prescriptions does not effectively enhance the probability of their adoption. Therefore, it is crucial to appropriately restrict and standardize the incentives for doctors' adherence to standardized practices within defined limits. This can help motivate medical practitioners to choose standardized practice strategies, encourage medical prescriptions to favor positive approaches for patients, and ultimately contribute to a reduction in instances of medical malpractice by patients.

**Figure 18:**
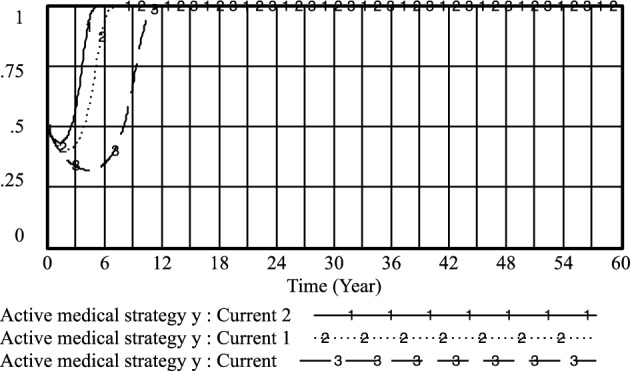
Effect of *U*_*1*_ and* R*_*1*_ on physician positive strategy selection.

**Figure 19:**
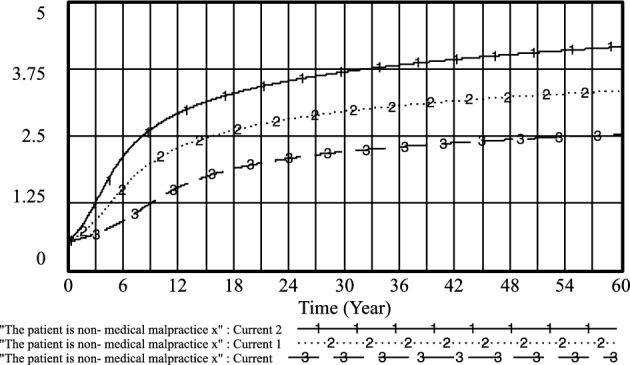
Effect of *U*_*1*_ and* R*_*1*_ on patients' selection of non-medical strategy.

By adapting patient perceptions and government regulation of patient medical behavior under the standardized practice of medical prescriptions, the following results were obtained: current: U_1_ = 0.1, R_1_ = 0.3; current 1: U_1_ = 0.3, R_1_ = 0.5; current 2: U_1_ = 0.5, R_1_ = 0.7. As shown in Figs. 18 and 19 that the higher the patient's cognition and the government's regulation of the patient's medical behavior under the standardized practice of medical prescriptions, the higher the likelihood of choosing an aggressive strategy, the earlier the time, and the higher the probability of the patient choosing the strategy of not treating the patient, the earlier the time. Therefore, improving the patient's awareness and the government's regulation of the patient's medical behavior under the standardized practice of medical prescriptions is conducive to improving the probability of the choice of active and cooperative strategies of the medical side and the choice of the patient's non-medical behavior strategy.

**Figure 20:**
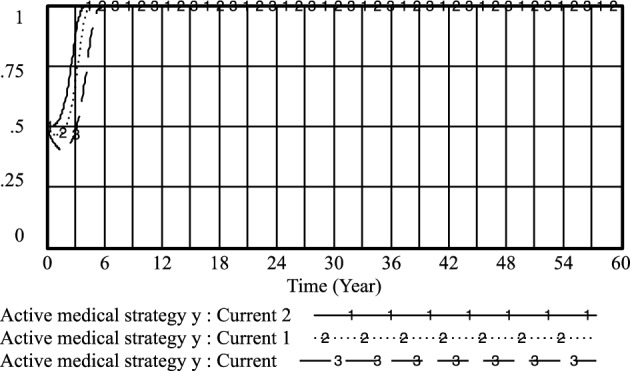
Effect of *PE*_*1*_ and* R*_*1*_ on physician positive strategy selection.

**Figure 21:**
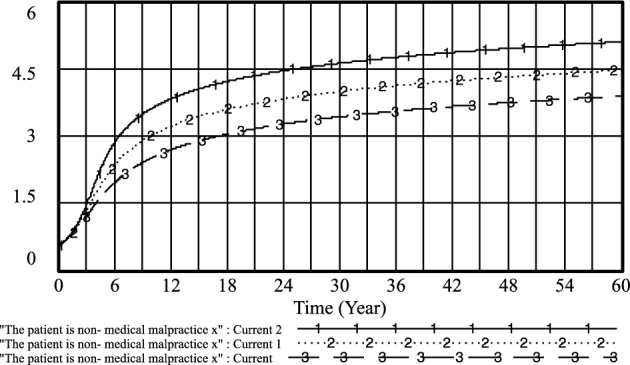
Effect of *PE*_*1*_ and* R*_*1*_ on patient's selection of non-medical strategy.

By adjusting the income of doctors when practicing under government regulations and the government's regulation of patients' medical behavior under standardized practice, the following results are obtained: *PE*_*1*_ = 0.4, *R*_*1*_ = 0.5; current 1: *PE*_*1*_ = 0.6, *R*_*1*_ = 0.7 and current 2: *PE*_*1*_ = 0.8, *R*_*1*_ = 0.9. As shown in Figs. 20 and 21, the higher the doctor's standardized practice income and the government's regulation of the patient's medical behavior under the standardized practice of medical prescriptions, the higher the likelihood of choosing an aggressive strategy, and the earlier the time of such choices. Additionally, the probability of patients choosing non-medical strategies also increases.

**Figure 22:**
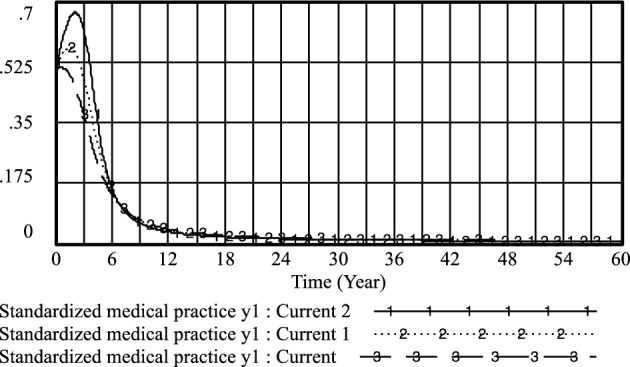
Effect of *PE*_*1*_ and* R*_*1*_ on physician standardized medical practice strategy selection.

However, it is observed that the probability of standardized practice initially increases with the increase of *PE*_*1*_ and *R*_*1*_, reaching a peak in the short term, but then decreases rapidly. Furthermore, with the increase of *PE*_*1*_ and the government's regulation of patients' medical behavior *R*_*1*_ under the standardized practice of medical prescriptions, the likelihood of medical prescriptions choosing standardized practice decreases. Moreover, *R*_*1*_ fluctuated little between 0.7 and 0.9, and the likelihood of selecting a standardized practice strategy for medical prescriptions remained almost unchanged. This indicates that when the government regulation is too high, it may not be conducive to the choice of standardized practice strategies by medical prescriptions, refer to Fig. 22.

In the long run, while improving the efficacy of doctors' standardized practice and the government's regulation of patients' medical behavior under the standardized practice of medical prescriptions can increase the likelihood of positive strategy choices by medical prescriptions and the selection of strategies for patients' non-medical behavior, it may not effectively improve the choice of standardized practice strategies by medical prescriptions.

## Discussion

### The critical influence of several factors on players' behavior in the evolutionary game

Utilizing an evolutionary game approach to examine medical malpractice within the context of information asymmetry and bounded rationality, we have developed a model for an evolutionary game between physicians and patients under state administration regulation. This model has been simulated by establishing dynamic equations and evolutionary stabilization strategies. It's important to note that the policy implications drawn from the conclusions are contingent upon the hypotheses posited in the model.

Our research findings highlight the critical influence of several factors on players' behavior in the evolutionary game, namely the advantages of doctors' standardized practice, the strength of government regulations, and patient perceptions. Based on these research findings, governmental actions aimed at addressing medical malpractice can be outlined as follows:

First, there is a need to adapt the regulatory framework. The government should reevaluate the suitability of the existing medical malpractice handling framework in the context of physician–patient disputes in China. This entails timely adjustments to the regulatory structure, tailoring the regulatory focus according to the unique characteristics of different provinces, cities, and years in which medical malpractice incidents occur. Diverse regulatory methods should be employed, and their application should vary based on multiple dimensions, including standardized and illegal practices by both doctors and patients, cooperation and conflict strategies, and patient-related medical issues. Such an approach would enhance the precision and effectiveness of regulation.

Second, the intensity of regulation concerning illegal medical practices and patient-related medical issues should be calibrated reasonably, with a diverse array of regulatory methods. The results of the evolutionary game demonstrate that, during the provision of medical services, medical practitioners weigh the benefits and costs to choose their strategies, as do patients in their medical treatment decisions. However, excessive regulatory force can discourage medical practitioners from selecting standardized practice strategies. In reality, excessive government regulation of medical behavior, particularly in relation to medical prescriptions, can reduce patients' medical trouble behavior but may weaken patient influence. This, in turn, may create an ideal practice environment for medical practitioners but is not conducive to the long-term development of medical services. It may increase the likelihood of medical practitioners resorting to illegal practices. Hence, striking a balance between regulation and safeguarding patient rights is essential to fostering harmonious physician–patient relationships.

Third, the establishment of an information management system for physicians and patients is crucial. Currently, there is a lack of publicly accessible individual medical malpractice statistics on the central government's official website in China. Therefore, it is imperative to construct a web-based information platform for healthcare incident prevention and management information services. This platform should enable both government and public access to real-time healthcare incident control. It would facilitate the immediate disclosure of medical malpractice cases, efficient management of the medical malpractice database, and a reduction in government regulatory costs.

Lastly, it's worth noting that doctor-patient game theory in government-regulated medical malpractice should also consider the influence of health insurance policies and the strategic choices of various stakeholders, including pharmaceutical companies. Consequently, a comprehensive analysis of diverse scenarios within the model remains a challenging endeavor. This study serves as a foundational exploration of the effective management of multi-party medical malpractice, laying the groundwork for further research in this domain.

## Methods

### Underlying assumptions and model construction

This study addresses two distinct subjects within the context of doctor-patient disputes under government regulation: healthcare practitioners and patients. Throughout the process of doctor-patient disputes, both parties make strategic choices under the umbrella of government regulations. In real-life scenarios, healthcare providers and patients exhibit bounded rationality, meaning they cannot possess complete information about each other's strategic choices due to information asymmetry. Consequently, both parties continually learn from successful strategies employed in prior doctor-patient disputes under government regulation. They adjust their own choices to maximize their benefits, ultimately reaching stable model outcomes. This aligns with the assumptions of bounded rationality, mutations, and selection principles in evolutionary game theory. Hence, evolutionary game theory proves to be a valuable tool for examining the strategic choices made by healthcare practitioners and patients in doctor-patient disputes under government regulation.

The evolutionary game model encompasses two groups of participants: physicians and patients. Physicians' behaviors are categorized as standardized practice or illegal practice, as well as cooperation or conflict. Patients' behaviors are divided into non-medical malpractice and medical malpractice. Standardized practice by physicians involves adhering to laws and regulations to ensure patient safety and treatment quality. In contrast, illegal practices by doctors encompass actions such as over-treating patients, prescribing expensive medications unnecessarily, failing to fulfill the duty of care, and delaying treatment, among others. Collaborative strategies involve physicians partnering with patients to facilitate cooperative treatment throughout the patient care continuum, emphasizing effective communication and well-planned treatment choices. Conversely, conflict strategies denote situations where cooperation breaks down. Patients' non-malpractice behavior signifies their choice not to engage in malpractice given the varying strategies employed by physicians.

As for the foundational assumptions of the model, while many studies often make direct assumptions, this study adopts a more grounded approach, drawing on previous research and aligning with real-world scenarios. Based on the Tort Liability Law of the People's Republic of China, the Regulations on the Prevention and Handling of Medical Malpractice, and other relevant laws and regulations, the fundamental assumptions of the model are outlined as follows:

#### Hypothesis 1

The proportion of physicians adopting standardized practice is denoted as *y*_*1*_, where 0 ≤ *y*_*1*_ ≤ 1, while the proportion engaging in irregular practice is represented as 1-*y*_*1*_. Similarly, the percentage of physicians opting for a collaborative strategy is expressed as *y*_*2*_, with 0 ≤ *y*_*2*_ ≤ 1, and those opting for a conflicting approach as 1-*y*_*2*_. For patients, the percentage not involved in medical malpractice is denoted as *x*, where 0 ≤ *x* ≤ 1, and those engaged in medical malpractice as 1-*x*. The likelihood of a physician selecting a conflict strategy is denoted as* y*. In the final evolutionary outcome, the physician chooses the strategy of "irregular practice and conflict practice", while the patient selects "non-medical malpractice" when the patient's payoff is positive, given the physician's choice of "irregular practice and conflict practice." Conversely, the probability of a physician choosing a conflict strategy is 1-*y.* In the final outcome, the physician opts for the strategy of "irregular practice and conflict practice", while the patient selects "medical malpractice" when the patient's payoff is negative, given the physician's choice of "irregular practice and conflict practice." The weights assigned to physicians' standardized practices and collaborative strategies in the physician–patient interaction are denoted as *S*_*1*_ and *S*_*2*_, respectively.

#### Hypothesis 2

Some hospitals may engage in illegal practices to maximize profits, often at the expense of patients. These unethical practices may include administering medically unnecessary treatments, prescribing excessively priced medications, and other unscrupulous tactics. The benefits derived from such practices surpass those under standardized practice conditions, *PE*_*2*_ > *PE*_*1*_. Furthermore, physicians may select different strategies, such as cooperation or conflict, when medical malpractice occurs, resulting in distinct outcomes. In healthcare, the cost of conflict between physicians and patients exceeds that of collaboration, *O*_*2*_ > *O*_*1.*_. However, the choice of different strategies—cooperation or conflict—between doctors and patients yields varying effects on the patient. In the medical service process, the benefits of cooperation between doctors and patients far outweigh those of conflict, *O*_*3*_ > *O*_*4*_.

#### Hypothesis 3

According to Article 54 of the Tort Liability Law of the People's Republic of China, when a patient suffers harm during medical diagnosis and treatment activities resulting from medical malpractice *H*, it constitutes a case of medical malpractice. In cases where the medical institution and its healthcare practitioners exhibit negligence, the medical institution becomes liable for compensating the incurred damages *D*. Concurrently, administrative authorities oversee and penalize any unlawful medical practices conducted by physicians, following the provisions of Article 47 of the Regulations on the Prevention and Handling of Medical Malpractice *M*. Many patients express dissatisfaction with the compensation received following their experiences with medical malpractice. If patients do not receive the anticipated compensation through the regular channels of legal defense, they may resort to pursuing additional compensation by employing a "medical malpractice" strategy. During the medical malpractice process, patients document their expenses related to transportation, lost wages, and other costs *E*. If a patient's "medical malpractice" behavior occurs within the framework of standardized medical prescriptions, it can lead to a more significant negative societal impact. This behavior may tarnish a physician's reputation, disrupt the normal order of diagnosis and treatment, and infringe upon other medical rights and interests. Consequently, the government department exercises stricter regulation over "medical malpractice" behavior in this scenario *R*_*1*_. Upon investigation, if it is determined that the healthcare practitioner has engaged in illegal practices, in such cases, even though the patient's "medical malpractice" conduct negatively influences the medical prescription, the patient also exposes the physician's violations during the process. As a result, the government exercises lesser control over "medical malpractice" behavior in this specific context *R*_*2*_, with *R*_*1*_ > *R*_*2*_.

#### Hypothesis 4

During instances of physician–patient medical malpractice, the patient's expectations regarding medical treatment strategies, treatment outcomes, treatment costs, and other factors influence their "medical malpractice" behavior, which is essentially their cognitive decision. If a patient holds high expectations for medical treatment strategies, treatment outcomes, treatment costs, etc., and possesses a heightened level of cognition, they are less likely to engage in "medical malpractice" behavior. This higher degree of cognition stems from their belief in the benefits generated by the doctor's treatment strategy *U*_*1*_, Conversely, if a patient has low expectations for medical treatment strategies, treatment outcomes, treatment costs, etc., indicating a lower degree of cognition, they are more prone to "medical malpractice" behavior. This is because their lower degree of cognition results in poor treatment outcomes and subsequently poorer perceived benefits, *U*_*2*_ > *U*_*1*_, leading to instances of "medical malpractice." A patient's "medical malpractice" behavior can diminish a physician's standing within the community, disrupt standard medical procedures, and potentially cause other forms of harm *L*_*1*_. For a physician who is not practicing within the confines of the law, a patient's "medical malpractice" behavior is likely to expose their illegal practices. Consequently, the physician may come under scrutiny from state regulatory bodies, the media, and various social groups, leading to heightened damages, *L*_*2*_, where *L*_*2*_ > *L*_*1*_. Furthermore, as "medical malpractice" incidents escalate, they not only disrupt the healthcare system but also have adverse repercussions on societal harmony and stability, the social status of physicians, and patient trust *k*.

Based on the aforementioned hypotheses, an evolutionary game payoff matrix for physicians and patients was constructed, as illustrated in Table 1.

**Table 1:** Doctors and patients under government regulation game payoff matrix.

Strategies		Payoffs	
P_1_	P_2_	Doctors	Patients
Non-medical malpractice *x*	Standardized practice *y*_*1*_, cooperation* y*_*2*_	*PE*_*1*_ − *O*_*1*_ − *D*	*D* − *H* + *O*_*3*_ + *kU*_*1*_
Non-medical malpractice *x*	Standardized practice *y*_*1*_, conflict 1 − *y*_*2*_	*PE*_*1*_ − *O*_*2*_ − *D*	*D* − *H* + *O*_*4*_ + *kU*_*1*_
Non-medical malpractice *x*	Illegal practices 1 − *y*_*1*_, cooperation* y*_*2*_	*PE*_*2*_ − *O*_*1*_ − *D*	*D* − *H* + *O*_*3*_ + *kU*_*1*_
Non-medical malpractice *x*	Illegal practices 1 − *y*_*1*_, conflict 1 − *y*_*2*_	*PE*_*2*_ − *O*_*2*_ − *D*	*D* − *H* + *O*_*4*_ + *kU*_*1*_
Medical malpractice 1 − *x*	Standardized practice *y*_*1*_, cooperation* y*_*2*_	*PE*_*1*_ − (*k* + 1)*D* − *kL*_*1*_ − *O*_*1*_	(*k* + 1)*D* − *k*(*E* + *R*_*1*_) − *H* + *O*_*3*_ − *kU*_*2*_
Medical malpractice 1 − *x*	Standardized practice *y*_*1*_, conflict 1 − *y*_*2*_	*PE*_*1*_ − (*k* + 1)*D* − *kL*_*1*_ − *O*_*2*_	(*k* + 1)*D* − *k*(*E* + *R*_*1*_) − *H* + *O*_*4*_ − *kU*_*2*_
Medical malpractice 1 − *x*	Illegal practices 1 − *y*_*1*_, cooperation* y*_*2*_	*PE*_*2*_ − (*k* + 1)*D* − *kL*_*2*_ − *M* − *O*_*1*_	(*k* + 1)*D* − *k*(*E* + *R*_*2*_) − *H* + *O*_*3*_ − *kU*_*2*_
Medical malpractice 1 − *x*	Illegal practices 1 − *y*_*1*_, conflict 1 − *y*_*2*_	*PE*_*2*_ − (*k* + 1)*D* − *kL*_*2*_ − *M* − *O*_*2*_	((*k* + 1)*D* − *k*(*E* + *R*_*2*_) − *H* + *O*_*4*_ − *kU*_*2*_

### Analysis of the evolutionary game between doctors and patients

#### Points of equilibrium in the evolutionary process

The average expected gains *EA*_*1*_ and *EA*_*2*_ for physicians choosing the standardized practice strategy and illegal practice strategy, the expected gains *EB*_*1*_ and *EB*_*2*_ for physicians choosing the collaborative strategy and the conflict strategy, as well as each physician's *E*$\overline{A}$,* E*$\overline{B}$ mean expected gain are:

1.1 $$\begin{aligned} EA_{1} &= x\left( {PE_{1} - O_{1} - D + PE_{1} - O_{2} - D} \right) + \left( {1 - x} \right)\left( {PE_{1} - \left( {k + 1} \right)D - kL_{1} - O_{1} + PE_{1} - \left( {k + 1} \right)D - kL_{1} - O_{2} } \right) \hfill \\ &= x\left[ {2kL_{1} + 2kD} \right] + 2PE_{1} - O_{1} - O_{2} - 2\left[ {kL_{1} + \, \left( {k + 1} \right)D} \right] \hfill \\ &= 2\left( {x - 1} \right)\left( {kL_{1} + kD} \right) - 2D + 2PE_{1} - O_{1} - O_{2} \hfill \\ \end{aligned}$$

1.2 $$\begin{aligned} EA_{2} &= x\left( {PE_{2} - O_{1} - D + PE_{2} - O_{2} - D} \right) + \left( {1 - x} \right)\left( {PE_{2} - \left( {k + 1} \right)D - kL_{2} - M - O_{1} + PE_{2} - \left( {k + 1} \right)D - kL_{2} - M - O_{2} } \right) \hfill \\ &= x\left( {2kL_{2} + 2kD + 2M} \right) + 2PE_{2} - O_{1} - O_{2} - 2kL_{2} - 2\left( {k + 1} \right){\text{D}} - 2M \hfill \\ &= 2(x - 1)(kD + M + kL_{2} ) + 2PE_{2} - 2D - O_{1} - O_{2} \hfill \\ \end{aligned}$$

1.3 $$\begin{aligned} \left( {E\overline{A}} \right) &= y_{1} EA_{1} + \left( {1 - y_{1} } \right)EA_{2} \hfill \\ &= \left( {y_{1} - 1} \right)\left( {2PE_{1} - 2PE_{2} + kL_{1} - kL_{2} + 2D - M} \right) - O_{1} - O_{2} + kD + 2PE_{1} + kL_{1} \hfill \\ \end{aligned}$$

1.4 $$\begin{aligned} EB_{1} &= x\left( {PE_{1} - O_{1} - D + PE_{2} - O_{1} - D} \right) + \left( {1 - x} \right)\left[ {PE_{1} - \left( {k + 1} \right)D - kL_{1} - O_{1} + PE_{2} - \left( {k + 1} \right)D - kL_{2} - M - O_{1} } \right] \hfill \\ &= x\left( {2kD + kL_{1} + kL_{2} + M} \right) + PE_{1} + PE_{2} - 2O_{1} + 2\left( {k + 1} \right)D - kL_{1} - kL_{2} - M \hfill \\ &= x\left( {x - 1} \right)\left( {2kD + kL_{1} + kL_{2} + M} \right) + PE_{1} + PE_{2} - 2O_{1} - 2D \hfill \\ \end{aligned}$$

1.5 $$\begin{aligned} EB_{2} &= x\left( {PE_{1} - O_{2} - D + PE_{2} - O_{2} - D} \right) + \left( {{1} - x} \right)\left[ {PE_{1} - \left( {k + {1}} \right)D - kL_{1} - O_{2} + PE_{2} - \left( {k + {1}} \right)D - kL_{2} - M - O_{2} } \right] \hfill \\ &= x\left( {{2}kD + kL_{1} + kL_{2} + M} \right) + PE_{1} + PE_{2} - 2O_{2} + {2}\left( {k + {1}} \right)D - kL_{1} - kL_{2} - M \hfill \\ &= x\left( {x - 1} \right)\left( {2kD + kL_{1} + kL_{2} + M} \right) + PE_{1} + PE_{2} - 2O_{2} - 2D \hfill \\ \end{aligned}$$

1.6 $$\begin{aligned} \left( {E\overline{B}} \right) &= y_{2} EB_{1} + \left( {1 - y_{2} } \right)EB_{2} \hfill \\ &= 2y_{2} \left( {O_{2} - O_{1} } \right) + x\left( {x - 1} \right)\left( {2kD + kL_{1} + kL_{2} + M} \right) + PE_{1} + PE_{2} - 2O_{2} - 2D \hfill \\ \end{aligned}$$

Let *y* denote the odds of a physician opting for a proactive strategy, and *S*_*1*_ and *S*_*2*_ denote the physician's weighting of the standardized practice and collaborative strategy on the relationship between the physician and patient, respectively.

$$\begin{gathered} y_{1} = S_{1} y_{1} \quad y_{2} = S_{2} y_{2} \hfill \\ Y = y_{1} /\left( {S_{1} + S_{2} } \right) + y_{2} /\left( {S_{1} + S_{2} } \right) \hfill \\ \end{gathered}$$

Therefore**,** F(*y*) = F(*y*_*1*_)/(*S*_*1*_ + *S*_*2*_) + F(*y*_*2*_)/(*S*_*1*_ + *S*_*2*_).

Following the Malthusian dynamic equation^32^, where an increase of the quantity of doctor standardized practice strategies is equivalent to *EA*_*1*_ subtracted from the average gain, and an increase of the quantity of doctor collaborative strategies is equivalent to *EB*_*1*_ subtracted from the average gain, with *t* being the time, this resolves the equation for the dynamics of physician replication:

1.7 $${\text{Let}}\,\,u = 2\left( {1 - x} \right)\left( {kL_{1} - kL_{2} - M} \right) + 2PE_{1} - 2PE_{2} \,\,{\text{then}}$$

1.8 $$F\left( y \right) = dy/dt = y\left[ {S_{1} u + S_{2} \left( {O_{2} - O_{1} } \right)} \right] + y_{2} \left[ { - S_{1}^{2} u - S_{2}^{2} \left( {O_{2} - O_{1} } \right)} \right]$$

Similarly, the expected benefits of *EC*_*1*_, *EC*_*2*_, and the mean beneficial effects of patients who choose non-medical strategy are:

1.9 $$\begin{aligned} EC_{1} &= y_{1} y_{2} \left( {D - H + O_{3} + kU_{1} } \right) + y_{1} \left( {{1} - y_{2} } \right)\left( {D - H + O_{4} + kU_{1} } \right) + \left( {{1} - y_{1} } \right)y_{2} \left( {D - H + O_{3} + kU_{1} } \right) + \left( {{1} - y_{1} } \right)\left( {{1} - y_{2} } \right)(D - H + O_{4} + kU_{1} ) \hfill \\ &= y_{2} \left( {O_{3} - O_{4} } \right) + D - H + O_{4} + U_{1} \hfill \\ \end{aligned}$$

1.10 $$\begin{aligned} EC_{2} &= y_{1} y_{2} [O_{3} + \left( {k + {1}} \right)D - H - kU_{2} - k\left( {E + R_{1} } \right)] + y_{1} \left( {{1} - y_{2} } \right)[\left( {k + {1}} \right)D - k\left( {E + R_{1} } \right) - H + O_{4} - kU_{2} ] + \left( {{1} - y_{1} } \right)y_{2} [O_{3} + \left( {k + {1}} \right)D - H - kU_{2} - k\left( {E + R_{2} } \right)] + \left( {{1} - y_{1} } \right) \, \left( {{1} - y_{2} } \right)[\left( {k + {1}} \right)D - k\left( {E + R_{2} } \right) - H + O_{4} - kU_{2} ] \hfill \\ &= ky_{1} \left( {R_{2} - R_{1} } \right) + y_{2} \left( {O_{3} - O_{4} } \right) + \left( {k + {1}} \right)D - H + O_{4} - U_{2} - k\left( {E + R_{2} } \right) \hfill \\ \end{aligned}$$

1.11 $$\begin{aligned} \left( {E\overline{C}} \right) &= yEC_{1} + ({1} - y)EC_{2} \hfill \\ &= x[y_{2} (O_{3} - O_{4} ) + D - H + O_{4} + U_{1} \left] { + \left( {{1} - x} \right)} \right[ky_{1} (R_{2} - R_{1} ) + y_{2} (O_{3} - O_{4} ) + \left( {k + {1}} \right)D - k(E + R_{2} ) - H + O_{4} - U_{2} ] \hfill \\ \end{aligned}$$

Equations for replication kinetics in patients:

1.12 $$F\left( x \right) = dx/dt = x\left( {{1} - x} \right)k\left[ {\left( {E - D + R_{2} } \right) - S_{1} y\left( {R_{2} - R_{1} } \right) + U_{1} + U_{2} } \right]$$

According to the two above equations, the two dimension power system (L) can be acquired.

1.13 $$\left\{ {\begin{array}{*{20}c} {F\left( x \right) = x\left( {{1} - x} \right)k\left[ {\left( {E + R_{2} - D} \right) - S_{1} y\left( {R_{2} - R_{1} } \right) + U_{1} + U_{2} } \right]} \\ {F\left( y \right) = y\left[ {S_{1} u + S_{2} \left( {O_{2} - O_{1} } \right)} \right] + y_{2} \left[ { - S_{1}^{2} u - S_{2}^{2} \left( {O_{2} - O_{1} } \right)} \right]} \\ \end{array} } \right.$$

In order to make it easier to analyze points of equilibrium and hence the systems' stability, make

1.14 $$y^{*} = S_{1} u + S_{2} \left( {O_{2} - O_{1} } \right)/S_{1}^{2} u + S_{2}^{2} \left( {O_{2} - O_{1} } \right),{ 0} < y^{*} < 1$$

##### Proposition 1

The system has equilibrium points A(0, 0), B(0, y*), C(1, 0), D(1, y*).

##### *Proof*

With respect to the two dimension kinetic system (L), *dy*/*dt* = 0 and *dx*/*dt* = 0, (0, 0), (0, y^*^), (1, 0), (1, y^*^) is the point of equilibrium of the system. Incorporating (0, y^*^) and (1, y^*^) into the system (L) also results in *dy*/*dt* = 0 and *dx*/*dt* = 0, obtaining the 4 points of equilibrium of system (L).

### Analysis of equilibrium points and stability

Evolutionary equilibrium point stability can be deducted from the local Jacobi stability analysis of the dynamical system^33^^.^ Consequently, the steady state of the equilibrium points is identified by the calculation of the characteristic values of the Jacobi matrices.

2.1a $${\text{J}} = \left[ {\begin{array}{*{20}c} {\partial F(x)/\partial x} & {\partial F(x)/\partial y} \\ {\partial F(y)/\partial x} & {\partial F(y)/\partial y} \\ \end{array} } \right]$$

2.1b $$= \left[ {\begin{array}{*{20}c} {a_{11} } & {a_{12} } \\ {a_{21} } & {a_{22} } \\ \end{array} } \right]$$

wherein, *a*_*11*_, *a*_*12*_, *a*_*21*_, *a*_*2*2_ are:

2.2 $$a_{11} = {\mathbf{\partial }}F(x)/{\mathbf{\partial }}x = \left( {{1} - {2}x} \right)k[\left( {D - E + R_{2} - S_{1} y\left( {R_{2} - R_{1} } \right) + U_{1} + U_{2} } \right]$$

2.3 $$a_{12} = {\mathbf{\partial }}F(x)/{\mathbf{\partial }}y = x\left( {{1} - x} \right)k[\left( {D - E + R_{2} - S_{1} \left( {R_{2} - R_{1} } \right) + U_{1} + U_{2} } \right]$$

2.4 $$a_{21} = {\mathbf{\partial }}F(y)/{\mathbf{\partial }}x = 0$$

2.5 $$a_{22} = {\mathbf{\partial }}F(y)/{\mathbf{\partial }}y = S_{1} u + S_{2} \left( {O_{2} - O_{1} } \right) + {2}y\left[ { - S_{1}^{2} u - S_{2}^{2} \left( {O_{2} - O_{1} } \right)} \right]$$

Equilibrium points for replicating dynamic equations are strategies for evolutionary stabilization that simultaneously satisfy the following two conditions^34^. (ESS).

2.6 $$\left( 1 \right)\,\,{\text{tr}}\,J = a_{11} + a_{22} < 0\,\,\left( {\text{Trace conditions}} \right);$$

2.7 $$\left( 2 \right)\det J = a_{11} a_{22} - a_{12} a_{22} > 0\,\,\,\left( {\text{Jacobi determinant condition}} \right).$$

Thus, 4 local equilibria *a*_*11*_, *a*_*12*_, *a*_*21*_ and a_22_**,** are attained (as shown in Table 2 below)**.**

2.8 $${\text{Thereinto}}y^{ * x = 0} = 2S_{1} (kL_{1} - kL_{2} - M + PE_{1} - PE_{2} ) + S_{2} \left( {O_{2} - O_{1} } \right)/2S_{1}^{2} (kL_{1} - kL_{2} - M + PE_{1} - PE_{2} ) + S_{2}^{2} \left( {O_{2} - O_{1} } \right)$$

2.9 $${\text{y}}^{{ * {\text{x}} = 1}} = 2S_{1} (PE_{1} - PE_{2} ) + S_{2} \left( {O_{2} - O_{1} } \right)/2S_{1}^{2} (PE_{1} - PE_{2} ) + S_{2}^{2} \left( {O_{2} - O_{1} } \right)$$

**Table 2:** The determinant and trace of the Jacobi matrix for the pointes *a*_*11*_* a*_*12*_* a*_*2*1_ and *a*_*22*_*.*

Equilibrium point	*a*_*11*_	*a*_*12*_	*a*_*21*_	*a*_*22*_
(0, 0)	*k*(*E* − *D* + *R*_*2*_ + *U*_*1*_ + *U*_*2*_)	0	0	2*S*_*1*_(*kL*_*1*_ − *kL*_*2*_ − *M* + *PE*_*1*_ − *PE*_*2*_) + *S*_*2*_(*O*_*2*_ − *O*_*1*_)
(0, y*)	*k*[*E* − *D* + *R*_*2*_ − *S*_*1*_y*^x = *0*^(*R*_*2*_ − *R*_*1*_) + *U*_*1*_ + *U*_*2*_]	0	0	− 2*S*_*1*_(*kL*_*1*_ − *kL*_*2*_ − *M* + *PE*_*1*_ − *PE*_*2*_) + *S*_*2*_(*O*_*2*_ − *O*_*1*_)
(1, 0)	− *k*(*E* − *D* + *R*_*2*_ + *U*_*1*_ + *U*_*2*_)	0	0	2*S*_*1*_(*PE*_*1*_ − *PE*_*2*_)
(1, y*)	− * k*[*E* − *D* + *R*_*2*_ − *S*_*1*_y*^x = *1*^(*R*_*2*_ − *R*_*1*_) + *U*_*1*_ + *U*_*2*_]	0	0	− 2*S*_*1*_(*PE*_*1*_ − *PE*_*2*_)

By determining the values of the determinants and traces of the Jacobi matrix, the determinant and trace values of the Jacobi matrix J can be obtained at the respective equilibrium points to determine the local stability.

1. When *D* > *E* + *R*_*2*_ + *U*_*1*_ + *U*_*2*_ and *S*_*2*_(*O*_*2*_ − *O*_*1*_)/2*S*_*1*_ + *kL*_*1*_ − *kL*_*2*_ − *M* < *PE*_*2*_ − *PE*_*1*_ < 0 or *D* > *E* + *R*_*2*_ + *U*_*1*_ + *U*_*2*_ and *PE*_*2*_ − *PE*_*1*_ > 0**,** the ESS of the system is (0, 0).
2. The ESS of the system is (0, y*) when *D* < *E* + *R*_*2*_ + *U*_*1*_ + *U*_*2*_ < *S*_*1*_y^*x=0^(*R*_*2*_ − *R*_*1*_) and *PE*_*2*_ − *PE*_*1*_ < *S*_*2*_(*O*_*2*_ − *O*_*1*_)/2*S*_*1*_ + *kL*_*1*_ − *kL*_*2*_ − *M*, or *S*_*1*_y^*x=0^(*R*_*2*_ − *R*_*1*_) < *E* + *R*_*2*_ + *U*_*1*_ + *U*_*2*_ < *D* and *PE*_*2*_ − *PE*_*1*_ < *S*_*2*_(*O*_*2*_ − *O*_*1*_)/2*S*_*1*_ + *kL*_*1*_ − *kL*_*2*_ − *M*.
3. When *D* < *E* + *R*_*2*_ + *U*_*1*_ + *U*_*2*_ and *S*_*2*_(*O*_*2*_ − *O*_*1*_)/2*S*_*1*_ < *PE*_*2*_ − *PE*_*1*_ < 0, or when *D* < *E* + *R*_*2*_ + *U*_*1*_ + *U*_*2*_ and *PE*_*2*_ − *PE*_*1*_ < *S*_*2*_(*O*_*2*_ − *O*_*1*_)/2*S*_*1*_, the ESS of the system is (1, y*).
4. When *D* < *E* + *R*_*2*_ + *U*_*1*_ + *U*_*2*_ and 0 < *PE*_*2*_ − *PE*_*1*_ < *S*_*2*_(*O*_*2*_ − *O*_*1*_)/2*S*_*1*_ + *kL*_*1*_ − *kL*_*2*_ − *M*, or when *D* < *E* + *R*_*2*_ + *U*_*1*_ + *U*_*2*_ and *PE*_*2*_ − *PE*_*1*_ < *S*_*2*_(*O*_*2*_ − *O*_*1*_)/2*S*_*1*_ + *kL*_*1*_ − *kL*_*2*_ − *M*, the ESS of the system is (1, 0).

According to the two-dimensional dynamical system in the paper, the partial instability of the Jacobi matrix J can be determined based on the values of each equilibrium point and determinant of the Jacobi matrix. Accordingly, on the basis of the values of the determinant and trace of the Jacobi matrix, it is shown in Tables 3 and 4 below for each of the two scenarios, namely, the first and the second scenarios, respectively. The methodology for determining the evolutionary stability of the other cases is consistent and is not repeated.

**Table 3:** The determinant and trace of the jacobi matrix for the pointes a_11_(0, 0).

Equilibrium point	*tr J*	*det J*	Stability
(0, 0)	−	+	ESS
(0, y*)	Unsure	−	Saddle point
(1, 0)	+	+	Unstable points
(1, y*)	Unsure	−	Saddle point

**Table 4:** The determinant and trace of the jacobi matrix for the pointes a_12_(0, y*).

Equilibrium point	*tr J*	*det J*	Stability
(0, 0)	Unsure	−	Saddle point
(0, y*)	−	+	ESS
(1, 0)	Unsure	−	Saddle point
(1, y*)	Unsure	−	Saddle point

### Supplementary Information

Supplementary Information.

## Data Availability

All data generated or analysed during this study are included in this published article (and its supplementary information files S1). Vensim PLE Version 6.4’s URL is https://vensim.com/software/#Vensim. Citespace V5.5 R2’s URL is http://cluster.cis.drexel.edu/~cchen/citespace/download/.
