# Supplementary material for: Evolutionary game theory and simulations based on doctor and patient medical malpractice under government regulation
Source: Sci Rep. 2023 Oct 25;13:18234. doi: 10.1038/s41598-023-44915-9 (PMC10600196; doi:10.1038/s41598-023-44915-9)
Supplement: Supplementary file 1 — Supplementary Information. [file 41598_2023_44915_MOESM1_ESM.zip › raw data /fig 1-2/project-no/CO-DESC_history_view_burstness_ByStartingYear.html]

View Citation Burst History

# Top 25 Keywords with the Strongest Citation Bursts

Keywords | Year | Strength | Begin | End | 2012 - 2022 || adverse event | 2012 | 3.48 | **2012** | 2013 | ▃▃▂▂▂▂▂▂▂▂▂ |
| medical error | 2012 | 3.08 | **2012** | 2013 | ▃▃▂▂▂▂▂▂▂▂▂ |
| medical education | 2012 | 2.55 | **2012** | 2015 | ▃▃▃▃▂▂▂▂▂▂▂ |
| professional liability | 2013 | 3.04 | **2013** | 2014 | ▂▃▃▂▂▂▂▂▂▂▂ |
| medical negligence | 2013 | 2.6 | **2013** | 2014 | ▂▃▃▂▂▂▂▂▂▂▂ |
| medical malpractice litigation | 2014 | 3.17 | **2014** | 2015 | ▂▂▃▃▂▂▂▂▂▂▂ |
| negligence | 2013 | 2.99 | **2014** | 2018 | ▂▂▃▃▃▃▃▂▂▂▂ |
| nerve | 2014 | 2.95 | **2014** | 2017 | ▂▂▃▃▃▃▂▂▂▂▂ |
| h index | 2014 | 2.64 | **2014** | 2015 | ▂▂▃▃▂▂▂▂▂▂▂ |
| legal responsibility | 2014 | 2.64 | **2014** | 2015 | ▂▂▃▃▂▂▂▂▂▂▂ |
| otolaryngology | 2015 | 3.82 | **2015** | 2018 | ▂▂▂▃▃▃▃▂▂▂▂ |
| disclosure | 2013 | 4.21 | **2016** | 2018 | ▂▂▂▂▃▃▃▂▂▂▂ |
| patient complaint | 2016 | 2.58 | **2016** | 2019 | ▂▂▂▂▃▃▃▃▂▂▂ |
| specialty | 2017 | 5.3 | **2017** | 2018 | ▂▂▂▂▂▃▃▂▂▂▂ |
| program | 2015 | 2.51 | **2017** | 2018 | ▂▂▂▂▂▃▃▂▂▂▂ |
| prevalence | 2016 | 3.35 | **2018** | 2019 | ▂▂▂▂▂▂▃▃▂▂▂ |
| resident | 2019 | 3.84 | **2019** | 2022 | ▂▂▂▂▂▂▂▃▃▃▃ |
| strategy | 2019 | 2.79 | **2019** | 2022 | ▂▂▂▂▂▂▂▃▃▃▃ |
| information | 2019 | 2.57 | **2019** | 2022 | ▂▂▂▂▂▂▂▃▃▃▃ |
| health care | 2016 | 4.18 | **2020** | 2022 | ▂▂▂▂▂▂▂▂▃▃▃ |
| mental health | 2020 | 2.87 | **2020** | 2022 | ▂▂▂▂▂▂▂▂▃▃▃ |
| time | 2020 | 2.87 | **2020** | 2022 | ▂▂▂▂▂▂▂▂▃▃▃ |
| diagnostic error | 2012 | 2.54 | **2020** | 2022 | ▂▂▂▂▂▂▂▂▃▃▃ |
| medical law | 2020 | 2.51 | **2020** | 2022 | ▂▂▂▂▂▂▂▂▃▃▃ |
| radiology | 2020 | 2.51 | **2020** | 2022 | ▂▂▂▂▂▂▂▂▃▃▃ |
