# Supplementary figures and images for: Evolutionary game theory and simulations based on doctor and patient medical malpractice under government regulation
Source: Sci Rep. 2023 Oct 25;13:18234. doi: 10.1038/s41598-023-44915-9 (PMC10600196; doi:10.1038/s41598-023-44915-9)

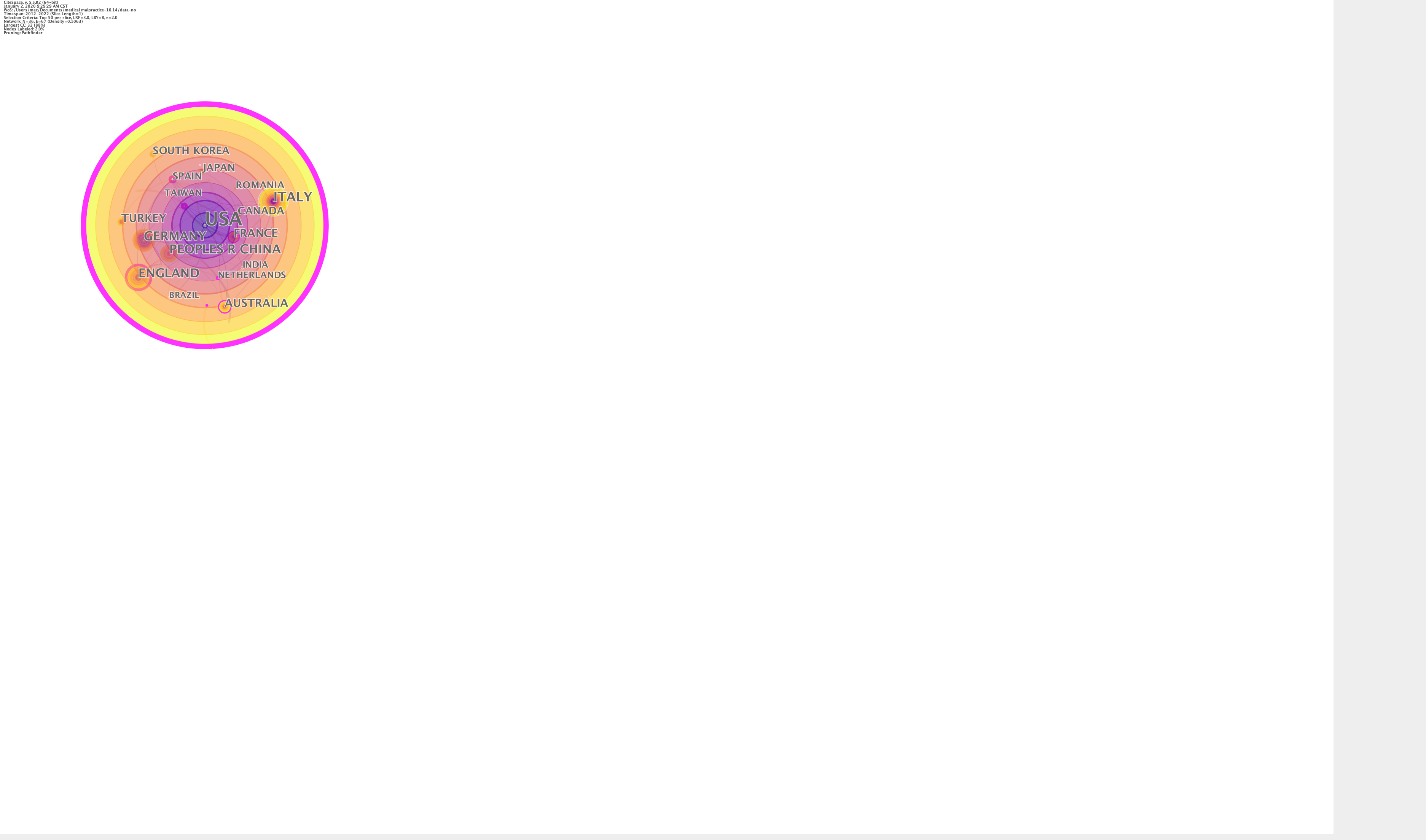

Supplement: Supplementary file 1 — Supplementary Information. [file 41598_2023_44915_MOESM1_ESM.zip › raw data /fig 1-2/project-no/fig.2.png]

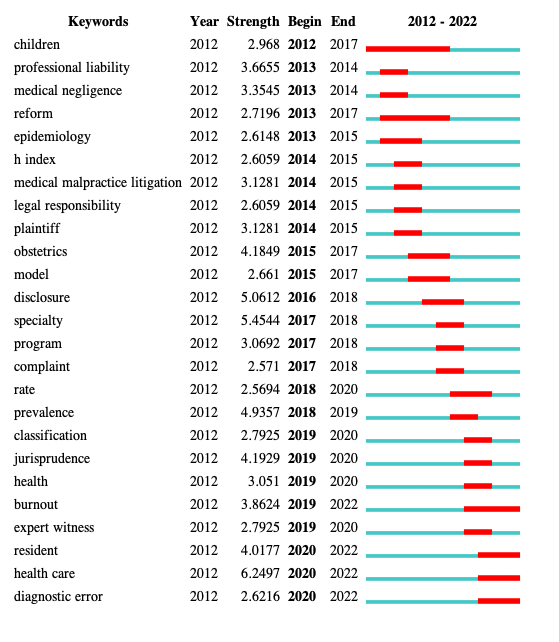

Supplement: Supplementary file 1 — Supplementary Information. [file 41598_2023_44915_MOESM1_ESM.zip › raw data /fig 1-2/project-no/fig.4.png]

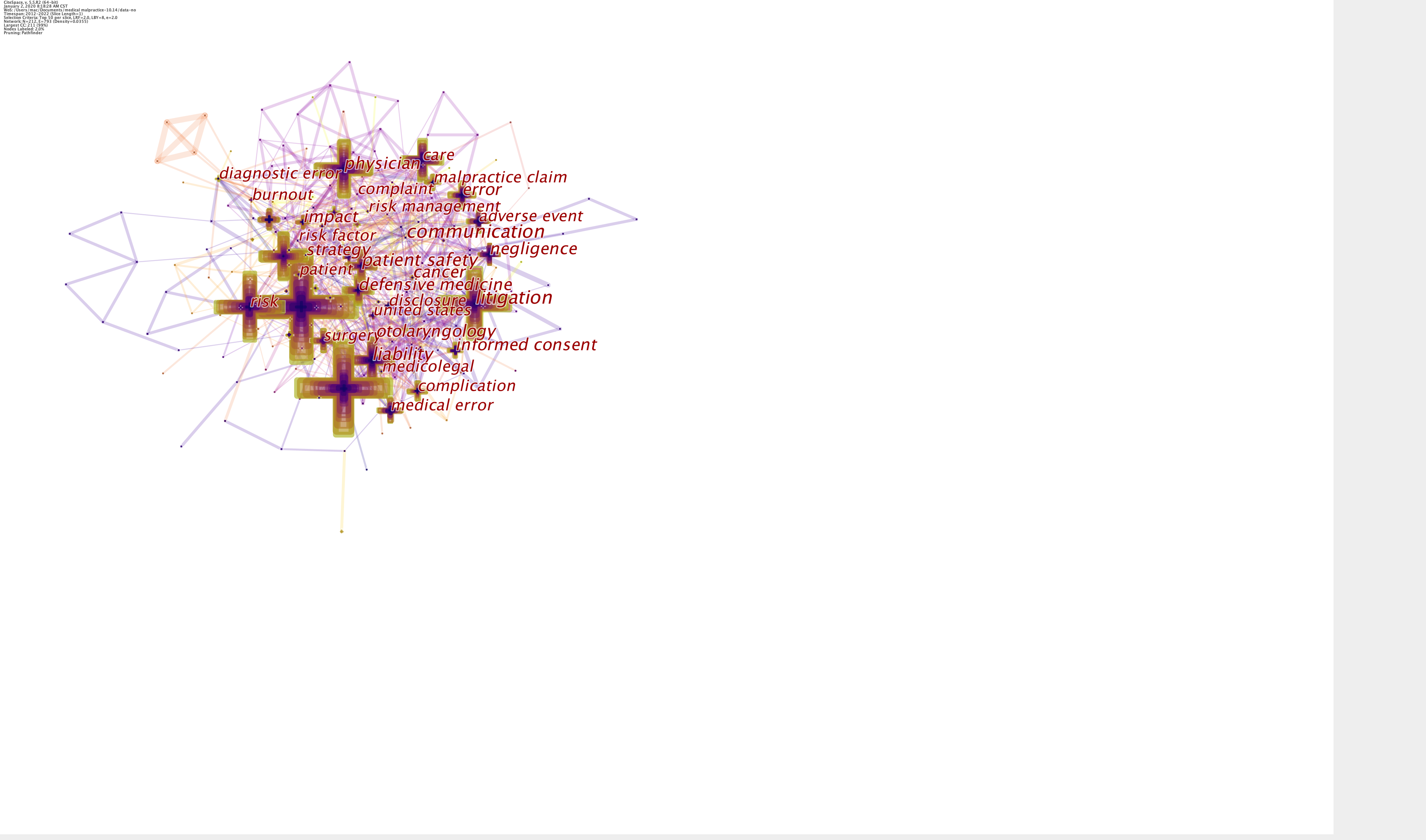

Supplement: Supplementary file 1 — Supplementary Information. [file 41598_2023_44915_MOESM1_ESM.zip › raw data /fig 1-2/project-no/fig.3.png]
